# Anomalous Right Coronary Artery from Left Main Coronary Artery and Subsequent Coursing between Aorta and Pulmonary Trunk

**DOI:** 10.1155/2013/195026

**Published:** 2013-12-17

**Authors:** Deephak Swaminath, Ragesh Panikkath, Jason Strefling, Alvaro Rosales, Roshni Narayanan, Jason Wischmeyer

**Affiliations:** Department of Internal Medicine, TX Tech Health Sciences Center, Lubbock, TX 79430, USA

## Abstract

Anomalous origin of left main coronary artery or right coronary artery from the aorta with subsequent coursing between the aorta and pulmonary trunk is rare and can be sometimes life threatening. After hypertrophic cardiomyopathy, coronary artery anomalies are the second most common cause of sudden cardiac deaths among young athletes. This is a case presentation of an anomalous origin of right coronary artery from left main coronary artery coursing between the pulmonary trunk and aorta. Patient presented with STEMI and had coronary bypass surgery.

## 1. Case Description

The patient is a 33-year-old male with an unremarkable past medical history and was transferred from an outside facility with an acute inferior myocardial infarction and ventricular tachycardia. Patient initially presented to the outside facility with complaints of chest pain. ECG showed lateral ST elevation and subtle inferior ST elevation. At the outside facility, patient developed ventricular tachycardia, and CPR was initiated with cardioversion 4 times before return of spontaneous circulation. The patient was subsequently intubated and transferred to our facility for further management.

**Figure 1:**
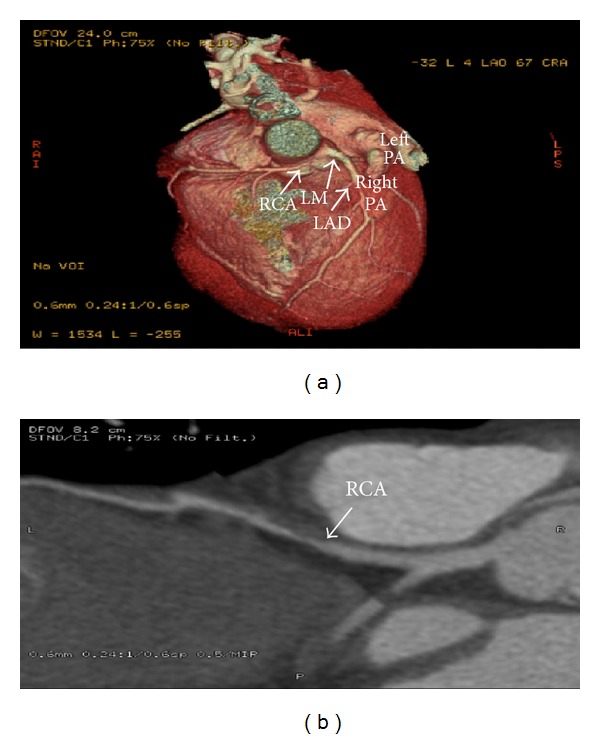
(a) Cardiac CT angiography with 3D reconstruction with digital subtraction of pulmonary arteries. (b) Cardiac CT angiography.

**Figure 2:**
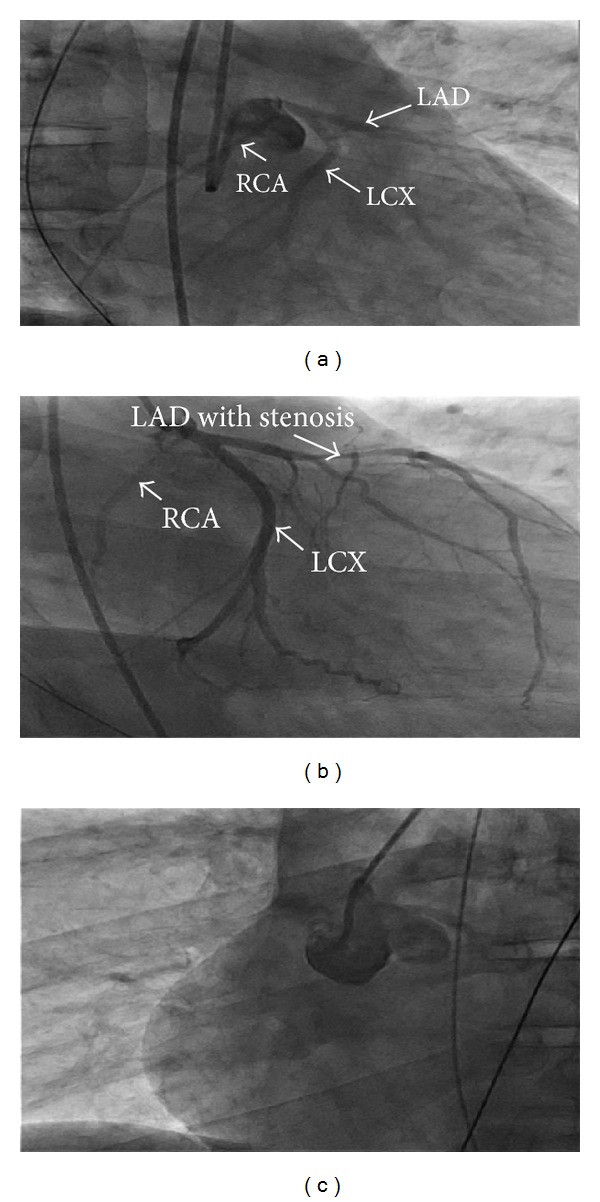
(a) Anomalous right coronary artery AP caudal projection with nonselective left coronary ostia angiogram. (b) Anomalous right coronary takeoff from left coronary artery in a RAO caudal projection of nonselective left coronary ostia angiogram. (c) Aortogram utilizing the JR4 catheter showing no separate right coronary ostium.

Upon transfer, the patient was taken for left heart catheterization (LHC) and selective coronary angiography. Left heart catheterization and angiography showed an 80% long tubular stenosis of the proximal to mid left anterior descending coronary artery (LAD) as well as an anomalous takeoff of the right coronary artery (RCA) from a left main coronary artery (Figures 2(a), 2(b), and 2(c)). LHC revealed markedly elevated left ventricular end diastolic pressure (LVEDP). The patient was aggressively diuresed after LHC revealed an elevated LVEDP. Dynamic ST changes improved with dual antiplatelet therapy, heparin, nitrates, and aggressive diuresis. Cardiac CT angiography with 3D reconstruction (Figures 1(a) and 1(b)) showed an anomalous origin of the right coronary artery coursing from the left main coronary artery between the aorta and pulmonary trunk. There was more than 70% narrowing at the origin of the right coronary artery from an apparent impingement from a plethoric pulmonary trunk. Cardiovascular surgery was consulted for surgical intervention. Aortocoronary bypass was performed with a left internal mammary artery bypass to the left anterior descending coronary artery and saphenous vein graft bypass to the right coronary artery. Postoperatively, the patient recovered and was discharged home.

## 2. Discussion

Coronary artery anomalies are found in 0.9%–1.3% of patients undergoing coronary angiography [1, 2]. Anomalous origin of the left main coronary artery or right coronary artery from the aorta coursing between the aorta and pulmonary trunk is rare and can be sometimes life-threatening. After hypertrophic cardiomyopathy, coronary artery anomalies are the second most common cause of sudden cardiac deaths among young athletes. Coronary artery anomalies are also the most common cause of nontraumatic sudden deaths in young American military recruits [2–4]. In a recent study, coronary artery anomalies were the cause of death in up to 19% of young athletes who died during or shortly after strenuous physical activity [3]. An interarterial course is clinically significant and carries a high risk for sudden cardiac death in young adults [5].

This is a case presentation of an RCA originating from the common left main coronary artery and coursing between the pulmonary trunk and aorta. The patient presented with ST segment elevation and life-threatening arrhythmia. Reduction of the right coronary blood flow secondary to impingement from a plethoric pulmonary trunk is likely attributed to the acute presentation. Notably, the patient did have anterolateral ST changes, suggestive of a hemodynamically significant LAD lesion, which may have contributed to the elevated LVEDP on presentation. In other presentations of compromising coronary anomalies, reduction in the coronary blood flow could be due to compression of the first segment of the coronary artery during its interarterial course, acute takeoffs, or slit-like orifices of these arteries. Anomalous coronary origin with interarterial coursing causes compromise in the coronary blood flow, which is evident during increases in pressure in the great vessels during exercise. Ischemic compromise of anomalous coronary arteries manifests as angina, syncope, congestive heart failure, arrhythmias, and sudden death [6–10].

However, coronary anomalies are often asymptomatic and might be discovered as an incidental finding. The physical exam, ECG, and stress test are generally unremarkable. An anomalous origin of a coronary artery and its route may be diagnosed by coronary angiography or more definitively by multislice computed tomography. Intravascular ultrasound provides high-resolution images to precisely evaluate coronary anomalies and luminal irregularity. The role of intravascular ultrasound was recently demonstrated in a small study that diagnosed an extrinsic compression of an anomalous coronary artery originating from the opposite sinus of Valsalva [11].

A study of two large registries composed of 27 young competitive athletes who died of sudden cardiac death (SCD) revealed that 23 had left main coronary artery from the right aortic sinus and 4 had a right coronary artery from the left sinus [12]. Estimates on rates of SCD with this condition come almost exclusively from autopsy data. Multiple studies focused on identifying specific factors that correlate with a higher likelihood of SCD in patient with coronary anomalies revealed that angle of take off, intramural course, slit-like ostium, interarterial course, vessel spasm, and intussusception of the anomalous vessel are the contributing factors [13]. However, in an attempt to identify specific features that were thought to contribute to risk of death, Taylor et al. looked at 30 pathology cases of anomalous coronary arteries and concluded that there were no anatomic features that could aid in risk assessment [14]. This assertion produces a quandary in risk assessment and likely means that these cases should be assessed individually on a case by case basis.

Anomalous origin of the right coronary artery from the left coronary sinus is 6 times more common than the contrary situation of the left coronary artery originating from the right coronary sinus. However, fortunately this presentation is believed to be more benign than its counterpart which is more malignant. Consequently, although surgical repair is advisable for all patients with the left coronary artery originating from the right coronary sinus, this is not the case with the right coronary artery originating from the left coronary sinus, where it might it be advisable to only a selected subgroup of patients [15]. The surgical options for anomalous coronary origins from contralateral sinus include bypass grafting, reimplantation of the anomalous vessel into its appropriate sinus, patch augmentation with or without pulmonary artery translocation, and unroofing the anomalous vessel [16]. Nonsurgical strategies include the use of beta blockers and avoidance of participation in all competitive sports for patients [15].
